# Virome of Giant Panda-Infesting Ticks Reveals Novel Bunyaviruses and Other Viruses That Are Genetically Close to Those from Giant Pandas

**DOI:** 10.1128/spectrum.02034-22

**Published:** 2022-08-02

**Authors:** Rui Ma, Min Zhao, Haoning Wang, Rong Hou, Kailin Qin, Yu Qian, Han Zhang, Yanshan Zhou, Wei Wu, Jiang Gu, Xiaochun Wang, Quan Shen, Songrui Liu, Jiabin Liu, Wenlei Bi, Xiang Yu, Shixing Yang, Feifei Feng, Zusheng Li, Long Zhang, Guanwei Lan, Chao Chen, Fei Xue, Yan Wang, Huang Chong, Yang Hong, Likai Ji, Yuwei Liu, Dunwu Qi, Tongling Shan, Wen Zhang

**Affiliations:** a Sichuan Key Laboratory of Conservation Biology for Endangered Wildlife, Chengdu Research Base of Giant Panda Breeding, Chengdu, Sichuan, China; b Department of Microbiology, School of Medicine, Jiangsu University, Zhenjiang, Jiangsu, China; c School of Geography and Tourism, Harbin University, Harbin, Heilongjiang, China; d Daxiangling Provincial Nature Reserve, Yaan, Sichuan, China; e Shanghai Veterinary Research Institute, Chinese Academy of Agricultural Sciences, Shanghai, China; Changchun Veterinary Research Institute

**Keywords:** ticks, tick-borne viruses, giant pandas, cross-species transmission, phylogenetic analysis

## Abstract

Tick infestations have been reported as one of the factors threatening the health of giant pandas, but studies of viral pathogens carried by ticks feeding on the blood of giant pandas are limited. To assess whether blood-sucking ticks of giant pandas can carry viral pathogens and if so, whether the viruses in ticks are associated with those previously detected in giant panda hosts, we determined the viromes of ticks detached from giant pandas in a field stocking area in Sichuan Province, southwest China. Using viral metagenomics we identified 32 viral species in ticks, half of which (including anellovirus [*n* = 9], circovirus [*n* = 3], and gemycircularvirus [*n* = 4]) showed homology to viruses carried by giant pandas and their associated host species (such as red pandas and mosquitoes) in the same living domain. Remarkably, several viruses in this study phylogenetically assigned as bunyavirus, hepe-like virus, and circovirus were detected with relatively high abundance, but whether these newly identified tick-associated viruses can replicate in ticks and then transmit to host animals during a blood meal will require further investigation. These findings further expand our understanding of the role of giant panda-infesting ticks in the local ecosystem, especially related to viral acquisition and transmission, and lay a foundation to assess the risk for giant panda exposure to tick-borne viruses.

**IMPORTANCE** Ticks rank only second to mosquitoes as blood-feeding arthropods, capable of spreading pathogens (including viruses, bacteria, and parasites) to hosts during a blood meal. To better understand the relationship between viruses carried by ticks and viruses that have been reported in giant pandas, it is necessary to analyze the viromes of giant panda-parasitic blood-sucking ticks. This study collected 421 ticks on the body surface of giant pandas in Sichuan Province, China. We characterized the extensive genetic diversity of viruses harbored by these ticks and reported frequent communication of viruses between giant pandas and their ticks. While most of the virome discovered here are nonpathogenic viruses from giant pandas and potentially tick-specific viruses, we revealed some possible tick-borne viruses, represented by novel bunyaviruses. This research contributes to the literature because currently there are few studies on the virome of giant panda-infesting ticks.

## INTRODUCTION

Tick and host population dynamics, warming climate, rapid urbanization, and political and socioeconomic alterations have increased human and other vertebrate exposure to tick infestation, thereby augmenting the infection rate of tick-borne viruses (TBVs) and incidence of tick-borne diseases (TBDs) in recent decades (1, 2). Tick infestation occurs in companion animals, livestock, and humans, as well as in wildlife such as the flagship species, giant pandas (3–8). Indeed, the blood-sucking ticks are common ectoparasites infesting wild and captive giant panda populations (9, 10). Their infestations can cause dermatitis, anemia, inflammation, exhaustion, and even death in giant pandas (7, 9). The hard tick infestation in giant pandas was first recorded in 1985 (10). Many more hard tick species have been reported to parasitize giant pandas since then, posing a continuing threat to their group development. The known records of ticks infesting giant pandas belonged to three genera of the family *Ixodidae*: *Ixodes*, *Haemaphysalis*, and *Dermacentor* (9).

By the end of 2020, the giant panda population at Chengdu Research Base of Giant Panda Breeding had reached 215, making it the world's largest captive-bred population of these creatures. As a branch of this panda base, the Daxiangling Reintroduction Base in Sichuan Province of southwest China is an experimental site for taking captive giant pandas back into the real natural environment. The free-roaming giant pandas living in the Daxiangling Reintroduction Base are commonly allowed free movement to search for water and food, increasing exposure to various ticks in the environment. Our previous research has elucidated the relationship of viruses in ‘Giant pandas-Associated animals-Arthropods’ and reported numerous cases of viral host-switching among these host species living in the same area (11). However, to date, the possible role of blood-sucking ticks acting as vectors participating in the circulation of some viruses from giant pandas and responsible for TBD spreading in this animal species is still ignored.

Up to now, more than 80 species of TBVs from six orders (*Bunyavirales*, *Mononegavirales*, *Asfuvirales*, *Amarillovirales*, *Articulavirales*, and *Articulavirales*) have been detected all over the world, some of which have been discovered to pose significant threats to animal and human health (12). For example, Crimean-Congo hemorrhagic fever virus (CCHFV, genus *Orthonairovirus*, family *Nairoviridae*) can circulate in nature in vertical and horizontal transmission cycles between ixodid ticks and animal hosts and develop viremia in infected hosts (13). When transmitted to humans, this virus can lead to a highly lethal hemorrhagic fever with a high case fatality ratio (up to 40%) (13–15). In recent years, the increased prevalence of tick-borne encephalitis virus (TBEV, genus *Flavivirus*, family *Flaviviridae*) has caused great concern (16). Rodents, insectivores, and birds can participate in the maintenance and circulation of TBEV in nature (17). In endemic areas, this zoonotic virus can be carried by the sentinel hosts—dogs (18). TBEV infection usually develops different disease manifestations in different animals (17). Powassan virus (POWV, genus *Flavivirus*, family *Flaviviridae*) is transmitted in natural cycles between mammalian wildlife hosts and hardbacked ticks predominantly of the genus *Ixodes* and can cause fatal neuroinvasive diseases in humans (19, 20). Taken together, many wild and domestic mammals are considered reservoirs and amplifying hosts of these TBVs, as they play an important role in supporting the tick vector population through blood-feeding and cofeeding transmission.

Despite concern over the possible emergence of TBV in giant pandas there remains little knowledge of virus diversity in giant panda-infesting ticks, or how viruses carried by them are related to those infecting giant pandas and even humans. Herein, we analyzed the viromes of blood-sucking ticks from the surface of giant pandas in the Daxiangling Reintroduction Base. With these results, we have not only characterized various novel tick-borne and tick-specific viruses but also discovered evidence for virus communication between the two hosts. This is important for future investigations of control of TBV infection in this vulnerable animal population.

## RESULTS

### Overview of tick virome.

**FIG 1:**
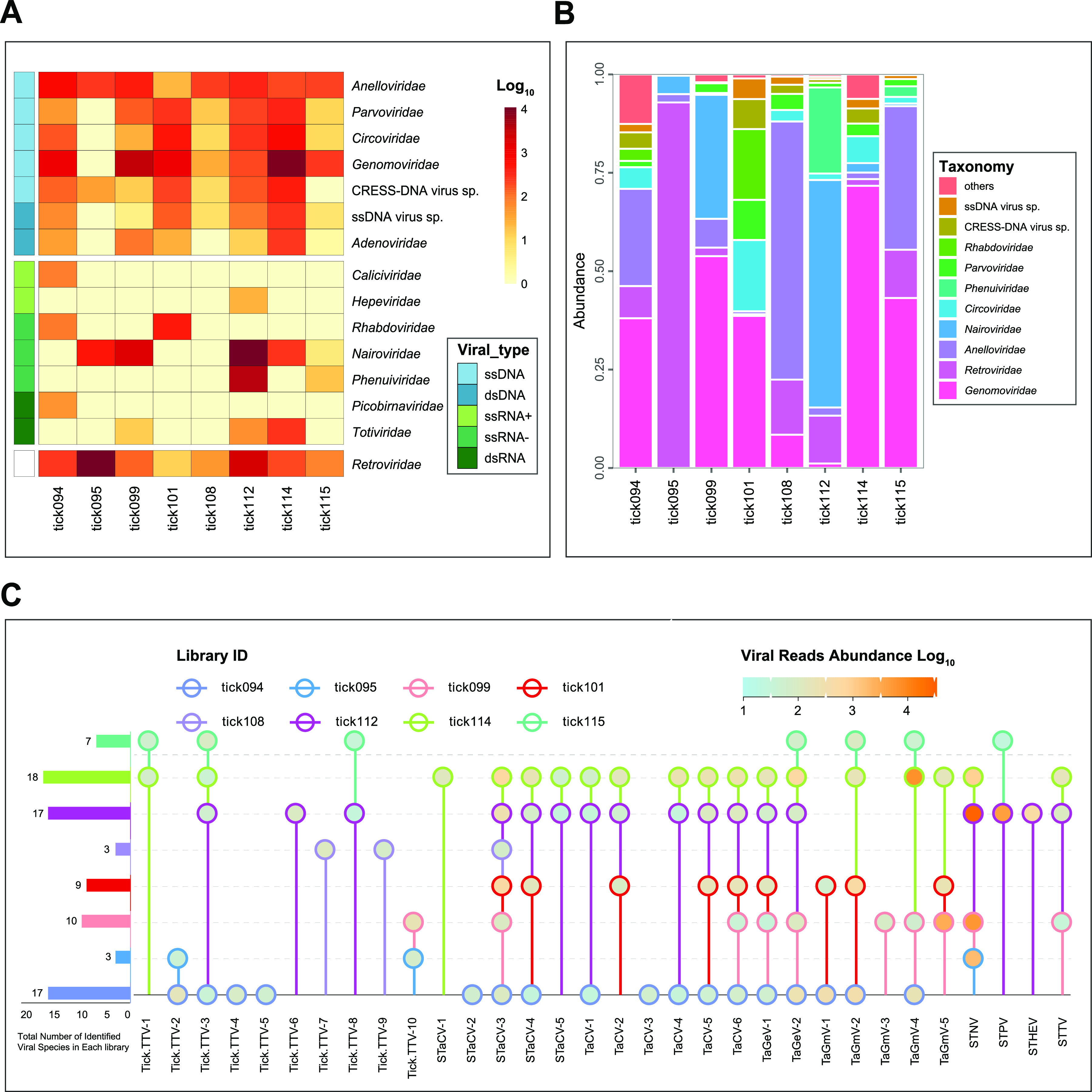
Taxonomic analyses of eukaryotic viral reads at the level of family or species. The heatmap (A) and bar graph (B) show the read counts of each viral family in each tick library. Viral types or viral families are shown with corresponding colors (see color legend). (C) The read abundance (node color) of each viral species. The viral species is indicated in the same color of node outline for each tick library.

In total, eight separate DNA libraries comprising 421 ticks collected from giant pandas were constructed and sequenced, resulting in a total of 60,332,534 of 250 base paired-end reads. Sequence reads were trimmed and *de novo* assembled within each barcode, and contigs and singletons were then compared to GenBank nonredundant protein database using BLASTx with an E-value cut-off 10^−5^. A total of 40 viral genomic sequences with significant similarity to known eukaryotic viruses were determined for further analysis (Table S1), among which 85% (34/40) were annotated to eukaryotic viruses spanning seven established families—*Anelloviridae* (*n* = 11), *Circoviridae* (*n* = 5), *Genomoviridae* (*n* = 11), *Nairoviridae* (*n* = 4), *Phenuiviridae* (*n* = 1), *Hepeviridae* (*n* = 1), and *Totiviridae* (*n* = 1)—while the remaining six genomes were assigned as the members of the unclassified CRESS-DNA viruses. A heatmap and bar graph were generated to investigate the taxonomic classification of eukaryotic viral reads at the family level (Fig. 1A and B). It is apparent that the viral abundance level for each pool varies considerably. The highest abundance was observed in library-tick112, whereas the lowest abundance occurred in library-tick108 (Fig. 1A). For read abundance of different viral families in each library, except library-tick095, -tick108, and -tick112, viral reads belonging to the families *Genomoviridae* had the most read counts (Fig. 1B). Furthermore, *Anelloviridae* and *Retroviridae* viruses had a quite abundant distribution in all libraries. Next, the mapping analysis using the 40 newly discovered genomes against the eight next-generation sequencing (NGS) data revealed the virus abundance and distribution in the eight tick pools (Fig. 1C). The most diverse virome was observed in library-tick114, followed by library-tick112 and -tick094; we detected sequences representing 18 and 17 viral species, respectively.

### Novel hepe-like and toti-like virus sequences.

**FIG 2:**
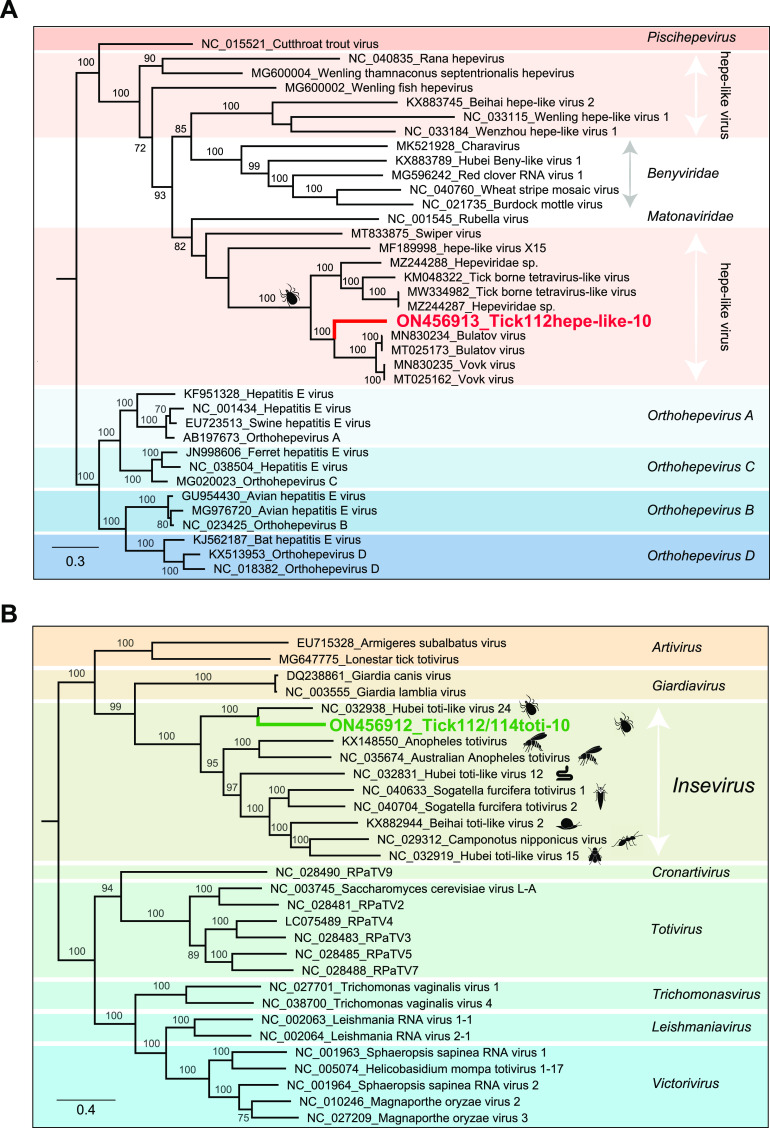
Phylogenetic analyses of STHEV and STTV sequences. (A) Bayesian phylogenetic tree for STHEV and other representative members from the families *Hepeviridae*, *Matonaviridae* and *Benyviridae* based on the amino acid sequences for RNA-dependent RNA polymerase protein (RdRp) domain. Nodes with bootstrap values ≥70 are indicated. (B) Bayesian phylogenetic tree for STTV and other representative members from the family *Totiviridae* based on the amino acid sequences for RdRp domain. Each scale bar indicates the amino acid substitutions per site.

Hepatitis E is a public health concern in several parts of Asia, Africa, the Middle East, and Mexico (21). Hepeviruses have been detected in fish, birds, and mammals (22). Recent research indicated that some hepe-like viruses are also carried by arthropods, including ticks and planthoppers (23, 24). Here, a novel Sichuan tick hepe-like virus (STHEV) strain Tick112hepe-like-10 was discovered in library-tick112. We only achieved a 5,302 nucleotide contig of this novel hepe-like virus that yielded a long open reading frame 1 (ORF1) in the standard genetic code. The amino acid (aa) sequence of the ORF1 nonstructural polyprotein (1,757 aa) in this virus was predicted to include three conserved domains: methyltransferase, RNA helicase, and RNA dependent RNA polymerase (RdRp) (Fig. S1), consistent with the genomic structure of other members in the family *Hepeviridae*. BLASTx search in GenBank indicated that Tick112hepe-like-10 had the best match of tick-derived Vovk virus and Bulatov virus (GenBank nos. MT025162 and MT025173), exhibiting 42.94% and 60.44% amino acid sequence identity across the single ORF and RdRp region, respectively. In the phylogenetic tree of RdRp, Tick112hepe-like-10 was grouped with tick-origin hepe-like viruses but formed a distinct branch, representing a novel clade in the family *Hepeviridae* (Fig. 2A). However, the epidemiological cycle, transmission route, and pathogenicity of these tick-associated hepe-like viruses remain obscure.

Similarly, we assembled a 2,489 nucleotide sequence with a partial ORF both in library-tick112 and -tick114, encoding for an incomplete nonstructural protein (773 aa) with the best match to the RdRp of Hubei toti-like virus 24 (hereinafter referred to as HTTV24), a sequence detected from ticks collected in China in 2013 (25). The novel sequence, tentatively named Sichuan tick toti-like virus (STTV), shared 47.74% nucleotide identity and 35.88% amino acid identity over the RdRp gene of HTTV24, representing a novel species in the family *Totiviridae*. In the RdRp protein phylogeny, STTV and HTTV24 grouped with insect totiviruses, which were assigned as an unapproved genus *Insevirus* (family *Totiviridae*) (Fig. 2B) (26).

### Novel bunyaviruses.

**FIG 3:**
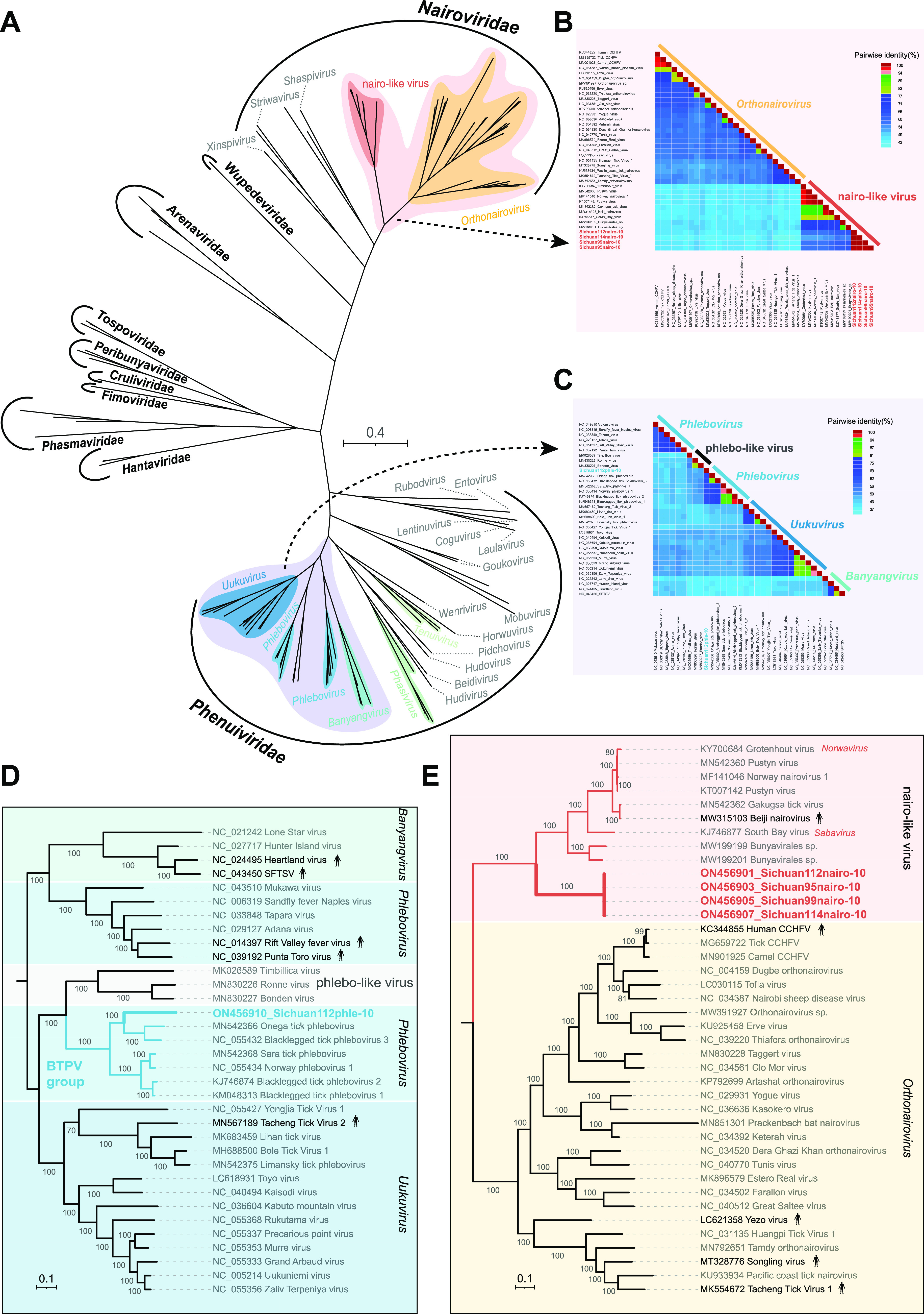
Phylogenetic relationship of *Bunyavirales*. (A) Phylogenetic tree based on RdRp domain amino acid sequences of the 10 families in the order *Bunyavirales*. (B) Pairwise genetic distance heatmap of *Orthonairovirus* and nairo-like virus RdRp domain protein sequences. (C) Pairwise genetic distance heatmap of *Phlebovirus*, *Banyangvirus*, *Uukuvirus*, and phlebo-like virus RdRp domain protein sequences. (D) Phylogenetic tree for STPV and members of the same or closely related genera in the family *Phenuiviridae*. (E) Phylogenetic tree for STNV, orthonairoviruses, and other nairo-like viruses in the family *Nairoviridae*. Each scale bar indicates the amino acid substitutions per site.

Among all negative-sense RNA viruses, 96% of the reads were related to the members in the order *Bunyavirales*, from which we assembled two novel virus sequences, tentatively named Sichuan tick nairovirus (STNV) and Sichuan tick phlebovirus (STPV). In the phylogeny of the RdRp domains of *Bunyavirales*, STNV and STPV belonged to the familiy *Nairoviridae* and *Phenuiviridae*, respectively (Fig. 3A). The mapping analysis using STNV and STPV genomes against the eight NGS data revealed that STNV was identified in four of the tick libraries (tick95, tick99, tick112, and tick114), and STPV occurred in two libraries (tick112 and tick115) (Fig. 1C). Members assigned to the family *Nairoviridae* and *Phenuiviridae* have a genome composed of three single-stranded negative-sense RNA segments: the large (L) segment, medium (M) segment, and the small (S) segment (27). Unfortunately, we were only able to assemble two segments of the two novel viral species: the large (L) segment encoding for RdRp and the small (S) segment encoding for nucleoprotein (4).

Pairwise amino acid comparisons showed that STNV and STPV shared less than 62% sequence identity with all other genetically related members in *Bunyavirales*, and hence, they represent two different novel species (Fig. 3B and C). The closest genetic relative to STPV is Onega tick phlebovirus (GenBank no. MN542366), a sequence detected from ticks sampled in Russia in 2018, and they shared 50.28% identity over their RdRp amino acid sequences and 41.43% identity over the nucleocapsid amino acid sequences. Meantime, the two phleboviruses were clustered within the Blacklegged tick phlebovirus (BTPV) group in the phylogenetic tree (Fig. 3D). Different from STPV, we assembled four highly similar genome sequences of STNV strains in different tick pools (99.50–99.71% nucleotide identities over the segment L and 99.65–99.88% nucleotide identities over the segment S). Phylogenetic analysis showed that STNV strains belonged to the nairo-like virus group, the closest sister cluster of the genus *Nairovirus* identified so far, but formed a monophyletic clade away from other nairo-like viruses, represented by South Bay virus (SBV) and Beiji nairovirus (BJNV) (Fig. 3E).

### Evidence for cross-species transmission of anelloviruses occurring during a blood meal of ticks.

**FIG 4:**
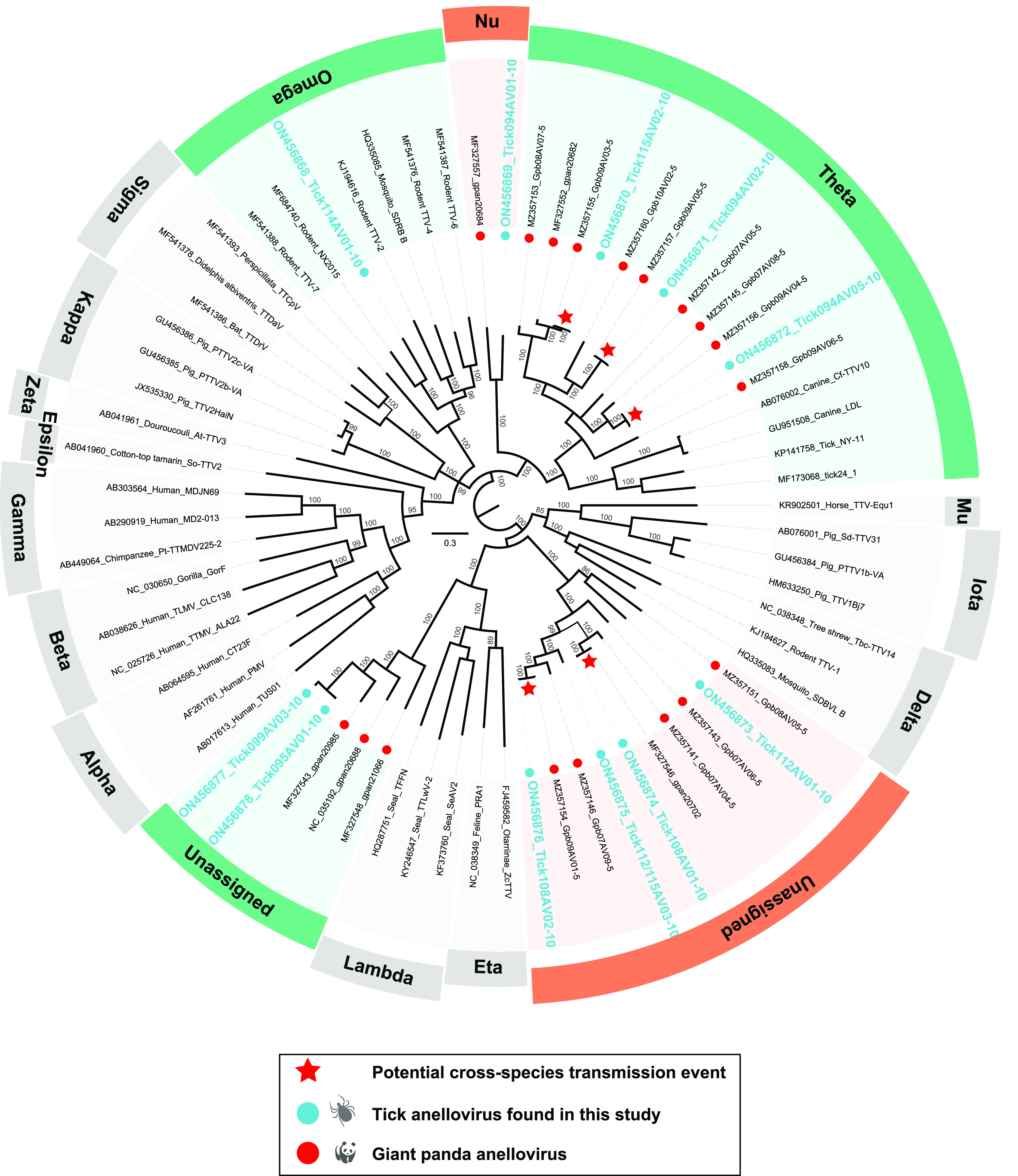
Phylogenetic analysis of the anelloviruses based on the ORF1 amino acid sequences. The newly identified viruses in ticks and previously identified in giant pandas are marked with blue and red dots, respectively. Putative cross-species transmission events between ticks and giant pandas are pointed with red pentagrams. Each scale bar indicates the amino acid substitutions per site.

Anelloviruses are small, nonenveloped, circular single-stranded DNA viruses. The anelloviruses have been detected in blood, saliva, semen, skin, genitourinary tract, gut, fecal, and nasopharyngeal swab samples from diverse hosts (28–30). In all tick pools, we obtained a total of 11 tick-associated anellovirus (Tick.TTV) sequences, which were highly divergent and clustered in five clades in the phylogenetic tree based on the ORF1 protein sequences of anelloviruses (Fig. 4). Remarkably, with the exception of Tick114AV01-10, all tick strains are most closely related to the Giant panda anellovirus (GpAV) previously detected in blood samples of giant pandas in Sichuan Province (from the sampling points adjacent to this study) (11, 28). We also recovered five Tick.TTV viruses that shared high degrees of identity (98.37%-100.00%) with GpAVs at protein level of the ORF1 (Fig. 4).

### Frequent cross-species transmission of CRESS-DNA viruses in ticks and potential reservoir host species.

**FIG 5:**
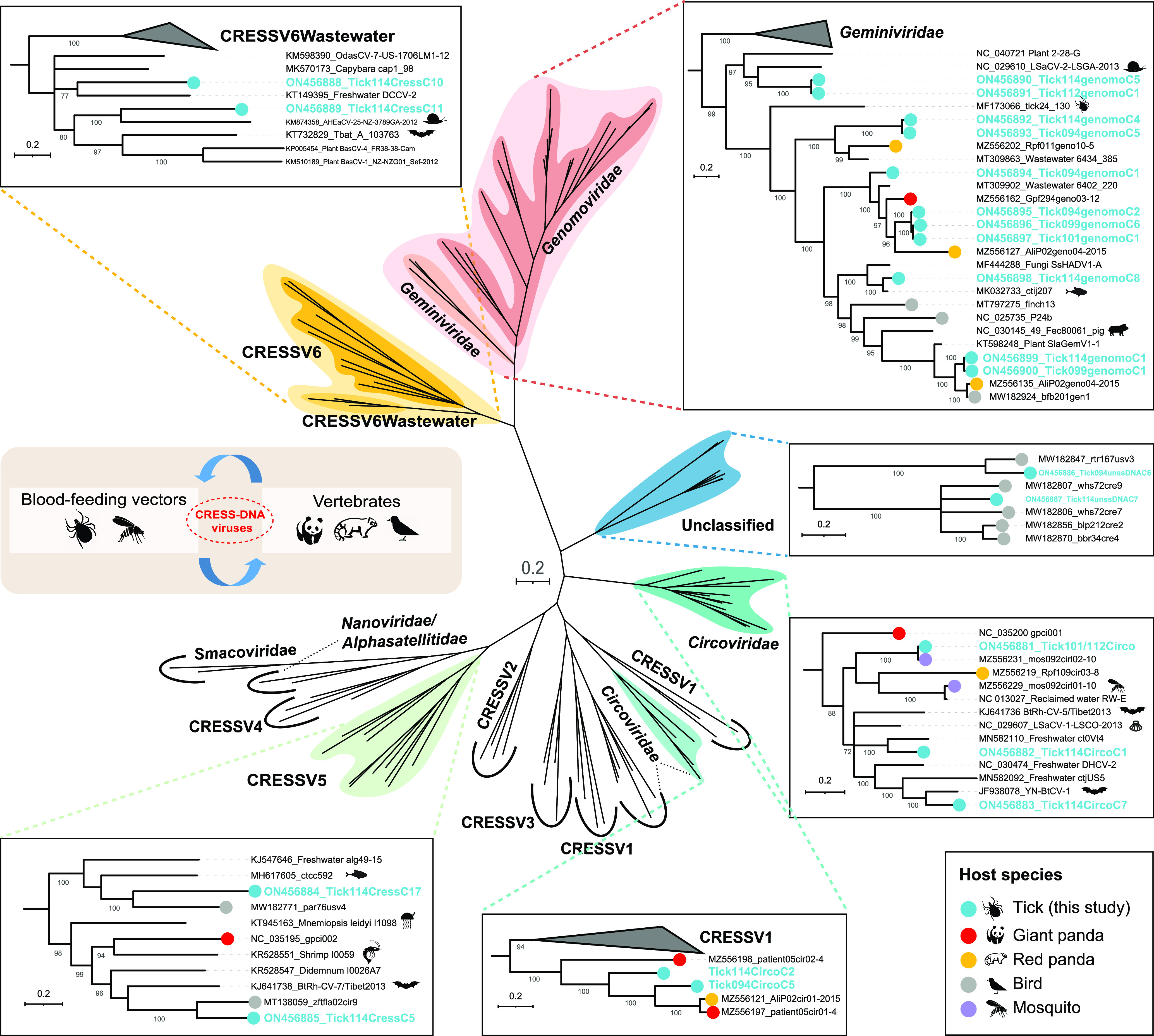
Phylogenetic analysis of the CRESS-DNA viruses based on the Rep amino acid sequences. The viruses identified from ticks in this study and other five different host species are marked with corresponding colors (see color legend). Each scale bar indicates the amino acid substitutions per site.

Currently, circular replication-associated protein (Rep)-encoding single-stranded DNA (CRESS-DNA) viruses are unified into seven classified families: *Genomoviridae*, *Geminiviridae*, *Circoviridae*, *Smacoviridae*, *Nanoviridae*, *Bacilladnaviridae*, and the newly established *Redondoviridae*, whereas many were assigned into unclassified groups (31, 32). Taxonomically, the Rep sequences of the 22 novel tick-derived CRESS-DNA virus strains described in this study split into the following groups: *Genomoviridae* (*n* = 11), *Circoviridae* (*n* = 5), CRESSV5 (*n* = 2), CRESSV6 (*n* = 2), and unclassified CRESS-DNA viruses (*n* = 2), exhibiting a high degree of genetic variability (Fig. 5). At an amino acid level, the Reps of 15 tick-associated CRESS-DNA viruses showed homology to that of the sequences from giant pandas, red pandas, wild birds, or mosquitoes sampled adjacent to this study (11, 28, 33). For example, within the circovirus group the Reps of two Sichuan tick-associated circoviruses (STaCV-1, strain Tick114CircoC2 and STaCV-2, strain Tick094CircoC5) were most closely related to that of a circovirus (GenBank no. MZ556121) previously identified in a red panda sample, sharing 50.18% and 63.46% amino acid identity, respectively. In particular, STaCV-3 was 100.00% identical to Sichuan mosquito circovirus 3 (GenBank no. MZ556231) at the nucleotide level of the Rep gene. And STaCV-3 was detected in all except library-tick115 and -tick095 (Fig. 1C). Further BLASTx analyses revealed that 13 of the 22 tick-associated CRESS-DNA viruses identified here shared <75% Rep amino acid sequence identities with their closest relatives.

## DISCUSSION

Ticks (class Arachnida, subclass Acari) are external parasites infesting a broad range of hosts, including reptiles, amphibians, mammals, and birds (12, 16). They can spread pathogens (including viruses, bacteria, and parasites) to hosts during a blood meal (3, 4, 34). According to Zhang et al. (35), 123 species of ticks have been identified in 1,141 locations across China from 1960 to 2017, accounting for >13% of the total tick species identified globally (20). And <10% of all tick species (over 900 species) are known to be essential in TBV and TBD transmission (16, 36).

Our previous research showed that the diversity of the virus community in giant pandas was high, and it showed high genetic similarity with a variety of viruses carried by other associated animal hosts in the same ecologic domain. Based on the limited evidence, we discussed the possible role of arthropod hosts in the communication of viral genetic information with vertebrate hosts (11). When added to our present work, the results further suggest that arthropods can act as vectors for vertebrate viruses interacting and exchanging genetic information, not by chance. This was apparent in two observations. The first key observation was the detection of 10 different tick-associated anellovirus genomes and that these viruses exhibited the highest similarity to previously detected GpAVs over ORF1 proteins. To our best knowledge, GpAVs are not associated with any known disease of pandas, which is similar to most other members in the family *Anelloviridae* (37). Our previous studies have revealed the extensive presence of different GpAV strains both in the blood samples of diseased and apparently healthy individuals (11, 28). The finding in this study provided direct evidence that viruses in the blood of giant pandas could be ingested by their ticks during a blood meal. The second observation was the presence of tick-associated gemycircularvirus 1 to 3 (TaGeV-1, 2, and 3), which shared the highest amino acid identities (78.80%–83.78%) in the Rep with that of the Giant panda feces-associated gemycircularvirus (GenBank nos. MF327569 and MZ556162). Meantime, the genomoviruses (GenBank nos. ON456899, ON456900, MZ556135, and MW182924) identified in the tick, red panda, and bird, respectively, were closely clustered together and shared a common ancestor. This data suggested that genomoviruses and even other CRESS-DNA viruses can be transported among ticks, mammals, and birds. In addition to exploring cross-species transmission of viruses, our work was aimed at uncovering viruses with potential relevance for diseases of mammals, and even humans.

In this study, 62,367 reads were assembled into 40 viral species in eight tick pools based on the most significant BLASTx similarities (E-value < 10^−5^). Generally, because of the multiple identical genomic sequences from the same virus existing in a single sample pool, the base mutation can be corrected by *de novo* assembly of the sequence reads from the deep sequencing data. As for some viral genomes with gaps based on the assembly results, we confirmed the full-length or nearly full-length genomic sequences by designing overlapping nested PCR (nPCR) primers followed by Sanger sequencing. To confirm the assembly results of a viral genome, reads had been *de novo* assembled back to the full-length genome using the low sensitivity/fastest parameter in Geneious Prime, where the abundance of sequence reads mapped against each target viral genome was analyzed and shown in Fig. 1C. Most of the assembled reads were attributed to STNV (60.64%, 37,821/62,367), followed by tick-associated gemycircularvirus 4 (11.77%, 7,339/62,367), and STPV (7.29%, 4,548/62,367). Of the 40 viral species identified here, only two novel species clustered phylogenetically with other arboviruses with potential relevance for vertebrate diseases: STNV fell within the family *Nairoviridae*, and STPV belonged to *Phenuiviridae*. Sichuan tick nairovirus fell phylogenetically within the nairo-like virus group but formed a monophyletic clade away from other nairo-like viruses, such as the pathogenic arbovirus, Beiji nairovirus. This finding further supports that the phylogeny of the family *Nairovirida*e is significantly more complex than previously appreciated (38). Similar to BJNV, all STNV strains missed the recognizable glycoprotein-coding segment (the medium segment), perhaps due to the high divergence or even the nonhomologous nature of these sequences (34). Interestingly, the four highly similar STNV strains were present in tick samples collected from two giant panda individuals on different dates. This finding revealed that STNV may be transovarially transmitted to progeny, resulting in the long-term prevalence of this virus in the local tick population. In addition to STNV, we identified a novel tick-borne phlebovirus, STPV. The complete sequence of the RdRp of this novel phlebovirus only shared 50.28% amino acid identity with its closest relative, satisfying the criteria for the definition of novel species within the genus *Phlebovirus* (39). There are examples of tick-borne viruses in the family *Phenuiviridae* which were shown to be pathogenic to humans, such as SFTS virus (genus *Banyangvirus*) (40), Heartland virus (genus *Banyangvirus*) (41), and Tacheng Tick Virus (genus *Uukuvirus*) (42). The members in the two genera *Banyangvirus* and *Uukuvirus* both have close phylogenetic relationships with the members within the genus *Phlebovirus* (Fig. 3D). However, the pathogenicity of tick-borne phleboviruses is currently still unclear.

This study has several limitations. Firstly, the ticks collected from giant pandas were only identified at the genus level based on cytochrome c oxidase subunit I gene sequences. Excluding *Ixodes* spp. ticks, the members of other tick genera were not pooled due to the limited number. Secondly, the study is limited in the number of sampled giant pandas and collection dates, and therefore, could not provide a better assessment of tick-borne virus pressure suffered by the local giant panda population. Lastly, our study is currently still at the stage of insight into the genetics and evolution of these novel viruses. The potential health impact of the newly identified viruses, such as bunyaviruses, on giant pandas, humans, and other mammals remains unknown and further experiments are required.

In sum, we have shown that the giant panda-infesting ticks harbor an extensive genetic diversity of viruses and share the common viral contents with giant pandas, largely due to communication of viruses between ticks and vertebrates. Notably, as all the giant pandas sampled appeared healthy, we cannot say definitively that the viruses identified here can cause disease in mammals, but it is clear that numerous viruses carried by ticks are genetically related to those found in giant pandas and other hosts living in the adjacent habitats.

## MATERIALS AND METHODS

### Sample collection and preparation.

In 2020, a total of 421 ticks were collected from two giant pandas living in Daxiangling Reintroduction Base (29°33'55.076″N—29°32'50.474″N, 102°50'13.866″E—102°51'3.189″E), the release experimental base of Chengdu Research Base of Giant Panda Breeding for captive giant pandas in Sichuan Province, China. The pandas were diagnosed as healthy and normal during routine monthly blood tests. Based on the trusting relationship with the staff, giant pandas were not hurt in the sample collection process. Sample collection was performed in the morning and all tick samples were taken from the ears, face, neck, and extremities of the giant pandas. These ticks were sampled by using labeled medical disposable sterile sampling kits, left on dry ice, and transported to the laboratory immediately. The tick species were identified by amplification and Sanger sequencing of the fragment of the cytochrome c oxidase subunit I (COI) gene. All the *Ixodes* spp. (Acari: Ixodidae) ticks were grouped into eight pools by the parasitic giant panda individual and sampling date (*n* = 50–55 ticks per pool; Table 1). Before resuspending in 1 mL Dulbecco’s Phosphate Buffered Saline (DPBS), ticks in each pool were homogenized, frozen, and thawed on dry ice three times and the supernatants were then collected after centrifugation (10 min; 15,000 × *g*; 4°C).

**TABLE 1:** Sampling information of ticks

Pool name	No. of ticks	Giant pandas^*a*^	Collection date
Tick094	50	HD	02-May-2020
Tick095	51	HD	03-May-2020
Tick099	52	HD	01-Jul-2020
Tick101	53	HD	03-Jul-2020
Tick108	53	QD	03-May-2020
Tick112	53	QD	01-May-2020
Tick114	54	QD	03-Jun-2020
Tick115	55	QD	01-Jun-2020

a The names of the two giant pandas are abbreviated.

### Library construction and next-generation sequencing.

Five hundred μL of each supernatant was filtered through a 0.45-μm filter (Millipore) to remove large cell-sized particles. The filtrate was incubated with DNase and RNase enzymes (Turbo DNase, Thermo Fisher Scientific, MA, USA; Baseline Zero DNase, Epicentre, WI, USA; Benzonase Nuclease, Novagen, MA, USA; and RNase A, Thermo Fisher Scientific) at 37°C for 60 min to degrade unprotected nucleic acid (28, 43–45). Total nucleic acids (total RNA and DNA) protected from nuclease digestion within viral capsids were extracted using the QiAamp Viral RNA minikit (Qiagen) under the manufacturer’s instructions. The viral nucleic acid samples were subjected to reverse transcription reactions using reverse transcriptase (Super-Script IV, Invitrogen) and 100 μmol of random hexamer primers, followed by a single round of DNA synthesis using Klenow fragment polymerase (New England BioLabs). Eight libraries were then constructed using Nextera XT DNA Sample Preparation Kit (Illumina) and sequenced on the Illumina NovaSeq 6000 platform.

### Bioinformatic analyses.

The generated 250-bp paired-end reads were debarcoded for each pool using vendor software from Illumina. Clonal reads were removed, and low sequencing quality tails were trimmed using Phred quality score 30 (Q30) as the threshold. The cleaned reads were then compared to an in-house nonvirus nonredundant (NVNR) protein database to remove false-positive viral hits using DIAMOND BLASTx search with default parameters (46). Then, taxonomic classification for DIAMOND results was parsed using MEGAN to perform the LCA-assignment algorithm according to default parameters. All viral sequence reads were *de novo* assembled using the Geneious Prime v2019.2.3 (Biomatters Ltd) (47). The contigs and singlet sequences were then matched against the viral proteome database using BLASTx (E-value < 10^−5^) (48) to confirm the virus types and remove false virus sequences. The open reading frames (ORFs) in the viral genome were predicted by combining Geneious Prime software and the BLASTx search results. Potential exons and introns were predicted by NetGene2 (https://services.healthtech.dtu.dk/service.php?NetGene2-2.42). The protein domains were identified and annotated using the NCBI conserved domain search (E-value < 10^−5^) (49).

### Phylogeny of viruses and data analysis.

All genome and protein sequence alignments were performed using the MEGA v10.2.5 program (50) with the default settings. The alignment results were manually checked using Geneious Prime. The Bayesian inference trees were then constructed using MrBayes v3.2.7 (51). We set ‘prset aamodelpr=mixed’ for the phylogenetic analysis based on the protein sequences, with two simultaneous runs of Markov chain Monte Carlo (MCMC) sampling in MrBayes. The runs were terminated until the standard deviation of the split frequencies <0.01, and the first 25% of trees were discarded as burn-in. Maximum-likelihood trees were also constructed to confirm all the Bayesian inference trees in MEGA software. The phylogenetic trees were visualized with Figtree v1.4.3 (http://tree.bio.ed.ac.uk/software/figtree/) and iTOL v6 (52). The heatmap presenting virus abundance in each library was constructed using the pheatmap package v1.0.12 (https://cran.r-project.org/package=pheatmap) based on the viral read number exported from Megan v6.18.9 (53) at the level of family in each barcode. Pairwise identity between the RdRp protein sequences of bunyaviruses was calculated using SDT v1.2 (54).

### Ethics statement.

All experimental protocols of this study were approved by the Institutional Animal Care and Use Committee of the Chengdu Research Base of Giant Panda Breeding (IACUC number 2020006).

### Data availability.

All raw data of NGS sequencing in this study were deposited in the NCBI Sequence Read Archive (SRA) database under the BioProject number: PRJNA808793. All virus genome sequences identified in this study have been deposited in GenBank under the accession numbers ON456868 to ON456913 (Table S1).
