# Supplementary material for: Virome of Giant Panda-Infesting Ticks Reveals Novel Bunyaviruses and Other Viruses That Are Genetically Close to Those from Giant Pandas
Source: Microbiol Spectr. 2022 Aug 2;10(4):e02034-22. doi: 10.1128/spectrum.02034-22 (PMC9430136; doi:10.1128/spectrum.02034-22)

SUPPLEMENTAL MATERIAL

Virome of giant panda-infesting ticks reveals novel bunyaviruses and other viruses that are genetically close to those from giant pandas

Rui Ma[1#], Min Zhao[2#], Haoning Wang[3#], Rong Hou[1], Kailin Qin[2], Yu Qian[2], Han Zhang[2], Yanshan Zhou[1], Wei Wu[1], Jiang Gu[1], Xiaochun Wang[2], Quan Shen[2], Songrui Liu[1], Jiabin Liu[1], Wenlei Bi[1], Xiang Yu[1], Shixing Yang[2], Feifei Feng[1], Zusheng Li[1], Long Zhang[1], Guanwei Lan[1], Chao Chen[1], Fei Xue[1], Yan Wang[2], Huang Chong[1], Yang Hong[4], Likai Ji[2], Yuwei Liu[2], Dunwu Qi[1]*, Tongling Shan[5]*, Wen Zhang[2]*

1. Sichuan Key Laboratory of Conservation Biology for Endangered Wildlife, Chengdu Research Base of Giant Panda Breeding, 610000 Chengdu, Sichuan, China;
2. Department of Microbiology, School of Medicine, Jiangsu University, 212013 Zhenjiang, Jiangsu, China;
3. School of Geography and Tourism, Harbin University, 150086 Harbin, Heilongjiang, China;
4. Daxiangling Provincial Nature Reserve, 625200 Yaan, Sichuan, China;
5. Shanghai Veterinary Research Institute, Chinese Academy of Agricultural Sciences, 200241 Shanghai, China.

[#]The authors contributed equally to this work.

[*] **Corresponding author:**

Wen Zhang, Email: z0216wen@yahoo.com;

Tongling Shan, Email: shantongling@shvri.ac.cn;

Dunwu Qi, Email: qidunwu@163.com.

TABLE S1 Detailed information of viral sequences identified in this study.

Accession no.	Virus strain name	Organism	Abbreviation	Taxonomy	Genome length (nt)	% GC content	Viral type	Best BLASTx hits on known protein of virus							
								Known protein	Accession no. of best match	Isolation/Strain	Country	Organism	% Coverage	E-value	% Identity
ON456868	Tick114AV01-10	Tick-assocaited anellovirus 1	Tick.TTV-1	Anelloviridae; Omegatorquevirus	1,446	47.3	ssDNA	ORF1	ATY37431	RtCe-AneV/NX2015	China	Anelloviridae sp.	84	1e-131	60.42
ON456869	Tick094AV01-10	Tick-assocaited anellovirus 2	Tick.TTV-2	Anelloviridae; Nutorquevirus	2,376	48.9	ssDNA	ORF1	ASH99133	gpan20684	China	Giant panda anellovirus	84	0	61.29
ON456870	Tick115AV02-10	Tick-assocaited anellovirus 3	Tick.TTV-3	Anelloviridae; Thetatorquevirus	1,914	41.1	ssDNA	ORF1	QZE11984	Gpb09AV03-5	China: Sichuan	Giant panda anellovirus	90	0	100.00
ON456871	Tick094AV02-10	Tick-assocaited anellovirus 4	Tick.TTV-4	Anelloviridae; Thetatorquevirus	1,720	41.1	ssDNA	ORF1	QZE11989	Gpb09AV05-5	China: Sichuan	Giant panda anellovirus	85	0	100.00
ON456872	Tick094AV05-10	Tick-assocaited anellovirus 5	Tick.TTV-5	Anelloviridae; Thetatorquevirus	2,069	43.4	ssDNA	ORF1	QZE11986	Gpb09AV04-5	China: Sichuan	Giant panda anellovirus	90	0	99.59
ON456873	Tick112AV01-10	Tick-assocaited anellovirus 6	Tick.TTV-6	Anelloviridae; unclassified Anelloviridae	1,690	44.6	ssDNA	ORF1	QZE11945	Gpb07AV04-5	China: Sichuan	Giant panda anellovirus	91	2e-107	50.94
ON456874	Tick108AV01-10	Tick-assocaited anellovirus 7	Tick.TTV-7	Anelloviridae; unclassified Anelloviridae	2,015	47.1	ssDNA	ORF1	ASH99099	gpan20702	China	Giant panda anellovirus	99	0	98.37
ON456875	Tick112/115AV03-10	Tick-assocaited anellovirus 8	Tick.TTV-8	Anelloviridae; unclassified Anelloviridae	1,433	45.6	ssDNA	ORF1	QZE11981	Gpb09AV01-5	China: Sichuan	Giant panda anellovirus	99	0	80.34
ON456876	Tick108AV02-10	Tick-assocaited anellovirus 9	Tick.TTV-9	Anelloviridae; unclassified Anelloviridae	1,763	46.6	ssDNA	ORF1	QZE11981	Gpb09AV01-5	China: Sichuan	Giant panda anellovirus	99	0	100.00
ON456877	Tick099AV03-10	Tick-assocaited anellovirus 10	Tick.TTV-10	Anelloviridae; unclassified Anelloviridae	2,086	46.9	ssDNA	ORF1	ASH99121	gpan21170	China	Giant panda anellovirus	89	0	63.54
ON456878	Tick095AV01-10	Tick-assocaited anellovirus 10	Tick.TTV-10	Anelloviridae; unclassified Anelloviridae	1,492	44.80	ssDNA	ORF1	ASH99121	gpan21170	China	Giant panda anellovirus	88	1e-178	66.75
ON456879	Tick114CircoC2	Sichuan tick-associated circovirus 1	STaCV-1	Circoviridae; Circovirus	2,484	40.7	ssDNA	Rep	UBJ26117	AliP02cir01-2015	China: Sichuan	Red panda circovirus 5	88	1e-98	50.18
ON456880	Tick094CircoC5	Sichuan tick-associated circovirus 2	STaCV-2	Circoviridae; Circovirus	2,178	42.0	ssDNA	Rep	UBJ26117	AliP02cir01-2015	China: Sichuan	Red panda circovirus 5	99	6e-134	63.46
ON456881	Tick101/112Circo	Sichuan tick-associated circovirus 3	STaCV-3	Circoviridae; Circovirus	2,979	41.8	ssDNA	Rep	UBJ25974	mos092cirl02-10	China: Sichuan	Sichuan mosquito circovirus 3	99	0	100.00
ON456882	Tick114CircoC1	Sichuan tick-associated circovirus 4	STaCV-4	Circoviridae; Circovirus	2,813	42.8	ssDNA	Rep	QGH73614	ct0Vt4	USA	CRESS virus sp. ct0Vt4	95	1e-134	72.93
ON456883	Tick114CircoC7	Sichuan tick-associated circovirus 5	STaCV-5	Circoviridae; Circovirus	1,084	39.8	ssDNA	Rep	AEL87784	YN-BtCV-1	China	Bat circovirus ZS/China/2011	99	6e-120	78.30
ON456884	Tick114CressC17	Tick-associated circular virus-1	TaCV-1	Cressdnaviricota; unclassified Cressdnaviricota	1,804	42.0	ssDNA	Rep	AXH77453	ctcc592	USA	Bacilladnavirus sp.	84	3.00E-61	41.51
ON456885	Tick114CressC5	Tick-associated circular virus-2	TaCV-2	Cressdnaviricota; unclassified Cressdnaviricota	2,123	48.6	ssDNA	Rep	QKN88878	zftfla02cir9	China	CRESS virus sp.	98	5e-144	66.45
ON456886	Tick094unssDNAC6	Tick-associated circular virus-3	TaCV-3	Cressdnaviricota; unclassified Cressdnaviricota	2,053	43.6	ssDNA	Rep	QTE03516	rtr167usv3	China	Luscinia sibilans CRESS-DNA-virus sp.	95	0	65.21
ON456887	Tick114unssDNAC7	Tick-associated circular virus-4	TaCV-4	Cressdnaviricota; unclassified Cressdnaviricota	2,736	58.2	ssDNA	Rep	QTE03533	blp212cre2	China	Pavo cristatus CRESS-DNA-virus sp.	87	9e-167	68.42
ON456888	Tick114CressC10	Tick-associated circular virus-5	TaCV-5	Cressdnaviricota; unclassified Cressdnaviricota	2,765	40.9	ssDNA	Rep	QDJ95229	cap1_98	Brazil	Capybara virus 11_cap1_98	90	1e-60	41.87
ON456889	Tick114CressC11	Tick-associated circular virus-6	TaCV-6	Cressdnaviricota; unclassified Cressdnaviricota	2,782	36.2	ssDNA	Rep	YP_009553348	OdasCV-7-US-1706LM1-12	USA	Odonata-associated circular virus-7	83	9e-54	38.78
ON456890	Tick114genomoC5	Tick-associated genomovirus 1	TaGeV-1	Genomoviridae; unclassified Genomoviridae	2,470	40.7	ssDNA	Rep	YP_009237554	LSaCV-2-LSGA-2013	New Zealand	Lake Sarah-associated circular virus-2	98	2e-89	49.50
ON456891	Tick112genomoC1	Tick-associated genomovirus 1	TaGeV-1	Genomoviridae; unclassified Genomoviridae	2,470	40.7	ssDNA	Rep	YP_009237554	LSaCV-2-LSGA-2013	New Zealand	Lake Sarah-associated circular virus-2	98	2e-89	49.50
ON456892	Tick114genomoC4	Tick-associated genomovirus 2	TaGeV-2	Genomoviridae; unclassified Genomoviridae	2,163	52.3	ssDNA	Rep	QJB18642	6434_385	USA	Genomoviridae sp.	99	4e-121	55.73
ON456893	Tick094genomoC5	Tick-associated genomovirus 2	TaGeV-2	Genomoviridae; unclassified Genomoviridae	2,163	52.6	ssDNA	Rep	QJB18642	6434_385	USA	Genomoviridae sp.	99	3e-120	55.73
ON456894	Tick094genomoC1	Tick-associated gemycircularvirus 1	TaGmV-1	Genomoviridae; Gemycircularvirus	2,182	50.9	ssDNA	Rep	ASH99172	gpge012	China	Giant panda associated gemycircularvirus	99	0	78.80

(continued on next page)

ON456895	Tick094genomoC2	Tick-associated gemycircularvirus 2	TaGmV-2	Genomoviridae; Gemycircularvirus	2,159	49.7	ssDNA	Rep	UBJ26249	Gpf294geno03-12	China: Sichuan	Giant panda feces-associated gemycircularvirus	99	0	83.78
ON456896	Tick099genomoC6	Tick-associated gemycircularvirus 3	TaGmV-3	Genomoviridae; Gemycircularvirus	2,057	50.0	ssDNA	Rep	UBJ26249	Gpf294geno03-12	China: Sichuan	Giant panda feces-associated gemycircularvirus	99	0	83.39
ON456897	Tick101genomoC1	Tick-associated gemycircularvirus 2	TaGmV-2	Genomoviridae; Gemycircularvirus	2,159	49.7	ssDNA	Rep	UBJ26249	Gpf294geno03-12	China: Sichuan	Giant panda feces-associated gemycircularvirus	99	0	83.78
ON456898	Tick114genomoC8	Tick-associated gemycircularvirus 4	TaGmV-4	Genomoviridae; Gemycircularvirus	2,176	53.0	ssDNA	Rep	QCW23688	ctij207	USA	Genomoviridae sp.	99	0	87.00
ON456899	Tick114genomoC1	Tick-associated gemycircularvirus 5	TaGmV-5	Genomoviridae; Gemycircularvirus	2,287	51.9	ssDNA	Rep	QVW56454	bfb201gen1	China	Emberiza spodocephala Genomoviridae sp.	99	0	86.26
ON456900	Tick099genomoC1	Tick-associated gemycircularvirus 5	TaGmV-5	Genomoviridae; Gemycircularvirus	2,286	52.0	ssDNA	Rep	QVW56454	bfb201gen1	China	Emberiza spodocephala Genomoviridae sp.	99	0	86.26
ON456901	Sichuan112nairo-10	Sichuan tick nairovirus	STNV	Nairoviridae; unclassified Nairoviridae	14,853	39.6	ssRNA-	RdRp	QVG74691	L1c	China: Yunnan	Bunyavirales sp.	100	0	67.11
ON456902	Sichuan112nairo-10	Sichuan tick nairovirus	STNV	Nairoviridae; unclassified Nairoviridae	3,755	47.4	ssRNA-	nucleocapsid protein	QPD01616	Rus/Ix_persulcatus/Karelia/1/2018	Russia	Gakugsa tick virus	86	5e-132	42.68
ON456903	Sichuan95nairo-10	Sichuan tick nairovirus	STNV	Nairoviridae; unclassified Nairoviridae	14,787	39.6	ssRNA-	RdRp	QVG74691	L1c	China: Yunnan	Bunyavirales sp.	100	0	67.11
ON456904	Sichuan95nairo-10	Sichuan tick nairovirus	STNV	Nairoviridae; unclassified Nairoviridae	3,586	47.9	ssRNA-	nucleocapsid protein	QPD01616	Rus/Ix_persulcatus/Karelia/1/2018	Russia	Gakugsa tick virus	86	5e-132	42.68
ON456905	Sichuan99nairo-10	Sichuan tick nairovirus	STNV	Nairoviridae; unclassified Nairoviridae	14,854	39.6	ssRNA-	RdRp	QVG74691	L1c	China: Yunnan	Bunyavirales sp.	100	0	67.11
ON456906	Sichuan99nairo-10	Sichuan tick nairovirus	STNV	Nairoviridae; unclassified Nairoviridae	3,704	47.5	ssRNA-	nucleocapsid protein	QPD01616	Rus/Ix_persulcatus/Karelia/1/2018	Russia	Gakugsa tick virus	86	5e-132	42.68
ON456907	Sichuan114nairo-10	Sichuan tick nairovirus	STNV	Nairoviridae; unclassified Nairoviridae	9,569	40.1	ssRNA-	RdRp	QVG74691	L1c	China: Yunnan	Bunyavirales sp.	100	0	67.11
ON456908	Sichuan114nairo-10	Sichuan tick nairovirus	STNV	Nairoviridae; unclassified Nairoviridae	3,490	39.3	ssRNA-	RdRp	ANT80542	SC1	USA	South Bay virus	87	1e-174	37.78
ON456909	Sichuan114nairo-10	Sichuan tick nairovirus	STNV	Nairoviridae; unclassified Nairoviridae	3,210	48.8	ssRNA-	nucleocapsid protein	QPD01616	Rus/Ix_persulcatus/Karelia/1/2018	Russia	Gakugsa tick virus	86	5e-132	42.68
ON456910	Sichuan112phle-10	Sichuan tick phlebovirus	STPV	Phenuiviridae; Phlebovirus	6,700	45.9	ssRNA-	RdRp	QPD01619	Rus/Ix_persulcatus/Karelia/3/2018	Russia	Onega tick phlebovirus	100	0	67.43
ON456911	Sichuan112phle-10	Sichuan tick phlebovirus	STPV	Phenuiviridae; Phlebovirus	1,642	50.9	ssRNA-	nucleocapsid protein	QPD01618	Rus/Ix_persulcatus/Karelia/3/2018	Russia	Onega tick phlebovirus	72	1e-51	41.43
ON456913	Tick112hepe-like-10	Sichuan tick hepe-like virus	STHEV	Hepeviridae; unclassified Hepeviridae	5,302	58.2	ssRNA+	RdRp	QKK82914	OTU62.IU3	Antarctica	Bulatov virus	100	2e-100	60.44
ON456912	Tick112/114toti-10	Sichuan tick toti-like virus	STTV	Totiviridae; unclassified Totiviridae	2,489	51.0	dsRNA	RdRp	YP_009336908	tick106628	China	Hubei toti-like virus 24	99	4e-78	50.61

FIG S1 Genomic structure, conserved domains or motifs of each viral species.

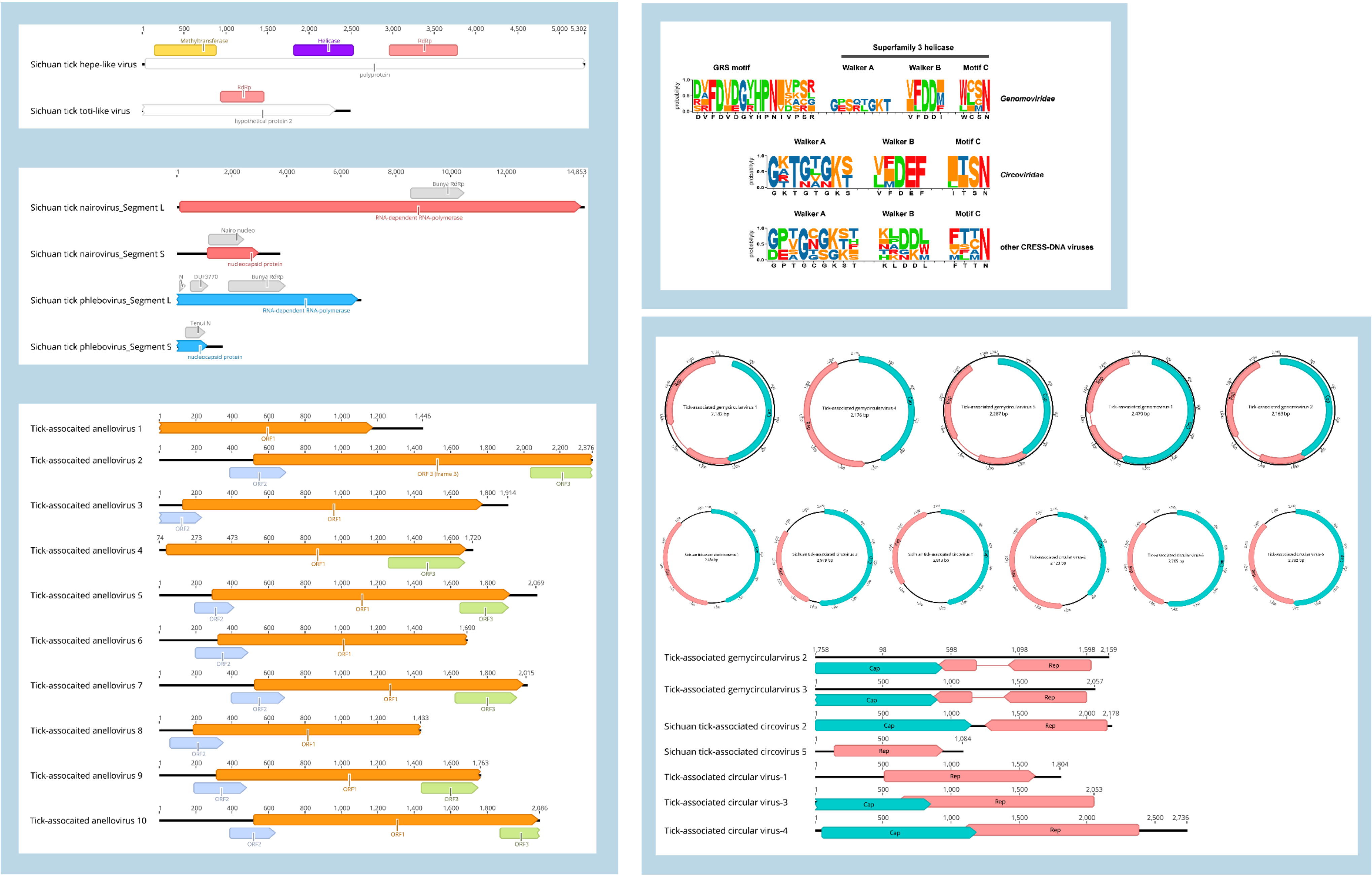

Supplement: Supplemental file 1 — Table S1 and Fig. S1. Download spectrum.02034-22-s0001.pdf, PDF file, 0.9 MB [file spectrum.02034-22-s0001.pdf]
